# p62/SQSTM1-induced caspase-8 aggresomes are essential for ionizing radiation-mediated apoptosis

**DOI:** 10.1038/s41419-021-04301-7

**Published:** 2021-10-25

**Authors:** Su Hyun Lee, Won Jin Cho, Abdo J. Najy, Allen-Dexter Saliganan, Tri Pham, Joseph Rakowski, Brian Loughery, Chang Hoon Ji, Wael Sakr, Seongho Kim, Ikuko Kato, Weon Kuu Chung, Harold E. Kim, Yong Tae Kwon, Hyeong-Reh C. Kim

**Affiliations:** 1 Department of Pathology, Wayne State University School of Medicine, Karmanos Cancer Institute, Detroit, MI 48201 USA; 2 Cellular Degradation Biology Research Center and Department of Biomedical Sciences, College of Medicine, Seoul National University, Seoul, 03080 Republic of Korea; 3 Department of Oncology, Wayne State University School of Medicine, Karmanos Cancer Institute, Detroit, MI 48201 USA; 4 Division of Radiation Oncology, Wayne State University School of Medicine, Karmanos Cancer Institute, Detroit, MI 48201 USA; 5 AUTOTAC Bio Inc., Changkkyunggung-ro 254, Jongno-gu, Seoul, 03080 Korea; 6 Department of Radiation Oncology, Kyung Hee University Hospital at Gangdong, College of Medicine, Kyung Hee University, Seoul, Korea; 7 SNU Dementia Research Center, College of Medicine, Seoul National University, Seoul, 03080 Republic of Korea; 8 Ischemic/Hypoxic Disease Institute, College of Medicine, Seoul National University, Seoul, 03080 Republic of Korea

**Keywords:** Cancer metabolism, Experimental models of disease

## Abstract

The autophagy–lysosome pathway and apoptosis constitute vital determinants of cell fate and engage in a complex interplay in both physiological and pathological conditions. Central to this interplay is the archetypal autophagic cargo adaptor p62/SQSTM1/Sequestosome-1 which mediates both cell survival and endoplasmic reticulum stress-induced apoptosis via aggregation of ubiquitinated caspase-8. Here, we investigated the role of p62-mediated apoptosis in head and neck squamous cell carcinoma (HNSCC), which can be divided into two groups based on human papillomavirus (HPV) infection status. We show that increased autophagic flux and defective apoptosis are associated with radioresistance in HPV(-) HNSCC, whereas HPV(+) HNSCC fail to induce autophagic flux and readily undergo apoptotic cell death upon radiation treatments. The degree of radioresistance and tumor progression of HPV(-) HNSCC respectively correlated with autophagic activity and cytosolic levels of p62. Pharmacological activation of the p62-ZZ domain using small molecule ligands sensitized radioresistant HPV(-) HNSCC cells to ionizing radiation by facilitating p62 self-polymerization and sequestration of cargoes leading to apoptosis. The self-polymerizing activity of p62 was identified as the essential mechanism by which ubiquitinated caspase-8 is sequestered into aggresome-like structures, without which irradiation fails to induce apoptosis in HNSCC. Our results suggest that harnessing p62-dependent sequestration of ubiquitinated caspase-8 provides a novel therapeutic avenue in patients with radioresistant tumors.

## Introduction

Crosstalk and interplay among programmed cell death and cell survival pathways play a critical role in normal cellular processes as well as under pathological conditions [1–4]. While apoptosis is most responsible for programmed cell death induced by developmental cues, intracellular damage or external death stimuli, autophagy is a major cell survival pathway that removes damaged cellular components and supports cellular metabolism [5, 6]. Autophagy has dual effects on cancer development and progression. It exerts tumor suppressive functions by removing damaged organelles and pathogens, contributing to the maintenance of genomic stability in normal cells [7]. However, as cancers progress, autophagy promotes cancer cell survival, thereby exerting oncogenic activity for cancer progression and therapy-resistance [8, 9]. Given that the impairment of the delicate interplay between autophagy and apoptosis is a contributing factor in the pathogenesis of many human diseases including cancers, efforts have been made to find the molecular and functional points of interactions between autophagy and apoptosis [10, 11]. In preclinical studies, pharmacological inhibition or genetic manipulation of autophagic regulators such as autophagy-related proteins (ATGs), mammalian target of rapamycin (mTOR), and phosphatidylinositol 3-kinase catalytic subunit type 3 (PI3KC3) have been shown to sensitize tumor cells to chemotherapy-induced cell death [9, 12, 13]. These studies have led to multiple clinical trials using autophagy inhibitors such as chloroquine or hydroxychloroquine as a strategy to promote cell death. Although this approach showed minor improvements in clinical trials, there are concerns about specificity and side effects associated with the systemic inhibition of autophagy [13, 14]. Thus, a critical need persists for the development of disease-specific and mechanism-based therapeutic tools to manipulate autophagic cell survival and programmed cell death.

The N-degron pathway is a proteolytic system wherein single N-terminal amino acids of proteins function as degradation determinants, called N-degrons, which are recognized by the recognition components (N-recognins) [15, 16]. The N-degrons include Arg, Lys, His (type 1; positively charged), Phe, Tyr, Trp, Leu, and Ile (type 2; bulky hydrophobic), as well as ATE1-mediated arginylation-permissive residues, exposed at the protein termini [17, 18]. The human genome encodes a set of N-recognins (UBR1, UBR2, UBR4, and UBR5) whose UBR-box domains bind N-degrons for substrate ubiquitination and proteasomal degradation [19, 20]. Recently, we found that the N-degron pathway also mediates autophagic proteolysis where the autophagic receptor p62/SQSTM1 acts as an N-recognin that binds N-degrons [21, 22]. In this process, the Nt-Arg residue of N-degrons binds the p62 ZZ domain to induce p62 self-polymerization and interaction with LC3 on autophagic membranes, leading to lysosomal degradation of p62-cargo complexes [21–23].

Paradoxical to the well-known function of p62 as an autophagic adaptor for cell survival, endoplasmic reticulum (ER) stress-induced apoptotic cell death was shown to occur through the ubiquitin-binding function of p62 leading to aggregation of ubiquitinated caspase-8 for subsequent proteolytic autoactivation of caspase-8 [24, 25]. The present study investigated the involvement of p62 in cancer progression and the feasibility of small molecule-mediated targeting of p62 for the therapeutic improvement in therapy-resistant cancer cells using head and neck squamous cell carcinoma (HNSCC) as a model.

HNSCC can be divided into two groups based on the status of human papillomavirus (HPV) infection [26–28]. Although the incidence of HPV( + ) head and neck cancer is rising, tobacco-induced carcinogenesis remains the leading cause of HNSCC [29–31]. HPV(-) HNSCC patients are less responsive to radiotherapy and have worse survival compared to HPV( + ) HNSCC patients [32–39]. At present, the mechanism of therapy-resistance in HPV(-) HNSCC is poorly understood. In the current study we found that an increased autophagic flux and apoptosis defects are associated with radioresistance in HPV(-) HNSCC, whereas HPV( + ) HNSCC fail to induce autophagic flux and readily undergo apoptotic cell death upon radiation treatments. We also show that the expression levels of cytosolic p62 proteins are associated with high grade tumors. These results suggest an association of cytoplasmic p62-mediated autophagy with disease progression and therapy resistance. Importantly, pharmacological activation of the p62 ZZ domain using synthetic small molecule ligands promote radiation-induced cytotoxicity via apoptosis induction in therapy-resistant HPV(-) HNSCC. We provide evidence that central to p62-dependent apoptosis in HPV(-) HNSCC is its self-oligomerization along with caspase-8 to form aggresomes-like complexes. Taken together, our study may pave the way for the development of a multimodal treatment to activate apoptosis in a p62-specific manner in intrinsically apoptosis-resistant cells.

## Materials and methods

### Cell culture

Human HNSCC cell lines UP-SCC-090 and UP-SCC-154 (established at University of Pittsburg), UM-SCC-19 (University of Michigan) and WSU-HN-12 (Wayne State University) were cultured as previously described [40].

### Antibodies and reagents

The antibodies used in this study are as follows: mouse monoclonal anti-p62 (Abcam, ab56416, 1:100,000), mouse monoclonal anti-FK2 specific to Ub-conjugated proteins (Millipore, 04-263, 1:1,000), rabbit polyclonal anti-LC3 (Sigma, L7543, 1: 10,000 for immunoblotting; 1:1,000 for immunofluorescence staining), anti-casapse-8 (Santa Cruz, sc-56070 1:2000 for immunoblotting, 1:200 for immunofluorescence staining), rabbit polyclonal anti-GAPDH (Santa Cruz, sc-25778, 1:2,000), rabbit polyclonal anti-GAPDH (BioWorld, AP0063, 1:20,000). The following secondary antibodies were used: alexa fluor 488 goat anti-rabbit IgG (Invitrogen, A11029, 1:200), texas red goat anti-mouse IgG (Invitrogen, T6390, 1:500), anti-rabbit IgG-HRP (Cell Signaling, 7074, 1:10,000), and anti-mouse IgG-HRP (Cell Signaling Technology, 7076, 1:10,000). Other reagents used in this study were bafilomycin A1 (Sigma); high capacity streptavidin agarose resin (Thermo Fisher Scientific).

### Ionizing radiation treatment (XRT)

As we previously described [41], cells were irradiated with 0, 2, 4, and 6 Gy using a gantry-mounted Best Theratronics Gammabeam 500 with a dose rate of 1 Gy/min. Irradiation was carried out at room temperature under atmospheric oxygen conditions. Delivered dose was confirmed with the use of a Farmer chamber.

### DEVDase activity assay

As we previously described [41], cells were lysed with a 0.5% NP40 lysis buffer, and 50 μg of protein lysates was incubated with 10 mmol/L Ac-DEVD-AMC substrate (Sigma) at 37 °C for 2 h. Fluorescence was detected using a SpectraMax Gemini (Molecular Probes, Carlsbad, CA) with 360 nm excitation and 460 nm emission.

### Establishment of p62-knockdown WSU12 cell line

Scrambled shRNA sequence (shScram; catalog no. RHS4346) and three shRNA against p62 GIPZ (shp62; clone ID no. V3LHS-375194, -375195, -375197) were obtained from Open Biosystems (Huntsville, AL). WSU12 cells were transfected with shScram or p62-targeting shRNA vectors using Lipofectamine 2000 (Invitrogen) and selected with 0.25 μg/mL puromycin. The resulting pooled population were referred to as shp62-94, shp62-95, and shp62-97, respectively.

### Immunofluorescence staining

Human HNSCC cells were cultured on cover slips and fixed with 4% paraformaldehyde in PBS (pH 7.4) for 15 min at room temperature. After washing three times with PBS, the cells were permeabilized with 0.5% Triton X-100/PBS solution for 15 min and washed three times with PBS for 5 min. After three washes with PBS, the cells were incubated with blocking solution (2% BSA in PBS) for 1 h and then with primary antibody overnight at 4 °C. Next day, the cells were washed five times for 10 min each time with PBS and then incubated with secondary antibody for 1 h. The cells were washed five times with PBS for 10 min each time, and DAPI stained for 10 min. After three washes with PBS, the coverslips were mounted on slides using the coverslips Fluoro-GEL (Electron Microscopy Sciences). Confocal images were taken by laser scanning confocal microscope 510 Meta (Zeiss) and analyzed using Zeiss LSM Image Browser (ver. 4.2.0.121).

### Immunoprecipitation assay

WSU12 cells, treated with vehicle control (DMSO) or YOK1104 for 12 h in the presence or absence of XRT (6 Gy), were lysed in binding buffer [20 mM Tris-HCl, pH 7.6, 125 mM NaCl, and 1% Nonidet P-40 with a cocktail of protease and phosphatase inhibitors (Roche)] through one cycle of freezing in liquid nitrogen and thawing in a 42 °C water bath. Genomic DNA was sheared by passing the extracts through a 26-gauge needle four times. The resulting lysates were centrifuged at 3000 × *g* for 15 min to pellet the cellular debris. Total 500 μg proteins were incubated with 2 μg control IgG or mouse monoclonal antibody raised against full-length recombinant caspase-8 of human origin (Santa Cruz, sc-56070) for overnight, followed by incubation with 40 μl of 50% protein A agarose bead slurry for 1 h. The beads were washed with the binding buffer for 5 min at 4 °C with gentle agitation; washing was repeated three times. The proteins bound to protein A/G agarose beads were dissociated in 2X SDS sample buffer, heated at 100 °C for 5 min, and separated on a SDS-PAGE. For immunoblot analysis of caspase-8 using caspase-8 IP products, the secondary HRP antibody (GeneTex, GTX221667-01), which specifically reacts with the native, non-reduced form of mouse IgG, was used to distinguish the caspase-8 band from immunoglobulin heavy chain bands.

### p62 immunohistochemistry

HNSCC blocks were retrieved from the Pathology core at Wayne State University and sections were deparaffinized and then hydrated. Microwave heat-induced antigen retrieval was performed using the Antigen Citrus Plus Retrieval Solution (BioGenex, HK081-5K). Non-specific binding sites were blocked with 2.5% normal horse serum in a wet chamber at 4 ^o^C, overnight. p62 (AbCam, ab56416) was diluted 1:2000 in blocking solution then incubated in a wet chamber at 4 ^o^C for 6 h. The ImmPRESS™ HRP Anti-Mouse IgG (Peroxidase) Polymer Detection Kit (Vector Laboratories, MP-7402) and the ImmPACT^TM^ DAB Peroxidase Substrate (Vector Laboratories, SK-4105) were used to develop the signal of stained slides followed by counterstaining with Mayer’s Hematoxylin (Scytek, HMM999). Images were obtained using a Zeiss Axioplan 2 microscope. Scoring was performed by a pathologist using a scale of 0 = no staining, 1 = low, 2 = moderate, 3 = high stain.

### Proximity Ligation Assay (PLA)

The proximity ligation assay was performed according to the manufacturer’s protocol using the Duolink™ In Situ Red Starter Kit Mouse/Rabbit (Sigma-Aldrich). Deparaffinization, hydration, and antigen retrieval were performed as described in the IHC section. p62 (AbCam, ab56416) and LC3 (Cell Signaling Technology, 2775) were diluted 1:2000 in antibody diluent overnight in a humidity chamber at 4 °C. Images were obtained using a Leica DMi3000 B Fluorescence Microscope.

### Statistical analysis

All experiments were repeated at least three times, and all data are presented as the mean ± SD. Statistical analysis was performed using Student’s unpaired two-tailed *t*-test or Chi-square test whose significance was determined according to *p* values (*p* < 0.001(***), *p* < 0.01(**), *p* < 0.05(*)).

## Results

### Activation of the autophagy–lysosome pathway is a marker of radioresistance in HPV(-) HNSCC

**Fig. 1:**
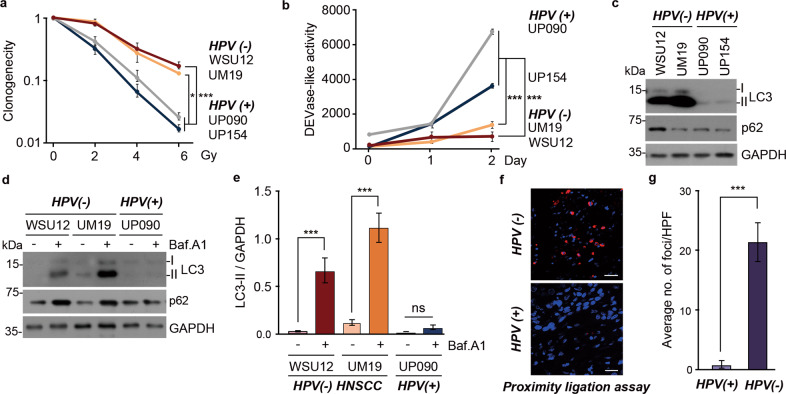
Defective apoptosis and increased autophagic flux in radioresistant HPV(-) HNSCC. **a** Clonogenic cell survival assay of HPV( + ) HNSCC cell lines (UP090 and UP154) and HPV(-) HNSCC (WSU12 and UM19) upon ionizing radiation (XRT) (*p < 0.05, ***p < 0.001). **b** DEVDase (caspase-3/caspase-7) activity assay in HNSCC cells at indicated time points post irradiation at 6 Gy (***p < 0.001). **c** Immunoblotting analysis of LC3 or GAPDH in HPV(-) HNSCC cell lines (WSU12 and UM19) and HPV( + ) HNSCC cell lines (UP090 and UP154). **d** Autophagic flux assay using WSU12, UM19 or UP154 cells in the absence or presence of bafilomycin A (500 nM, 6 h). **e** Quantification of LC3-II band intensity of **d**. Error bars represent means ± SD from three independent experiments (***p < 0.001, ns: non-significant). **f** In situ detection of autophagosomes in HPV(-) and HPV( + ) HNSCC tissues by p62 and LC3 PLA. Scale bar: 30 μm. **g** The average number of positive foci in p62 and LC3 PLA quantitated at 10X magnification (*n* = 4, ***p < 0.001).

To understand the molecular mechanisms of radioresistance in HNSCC cells, we assessed the relationship between autophagic activities and radiosensitivity in HPV(-) HNSCC cells (WSU12 and UM19) compared to HPV( + ) HNSCC cells (UP090 and UP154). The HPV status in these cell lines was confirmed by RT-PCR analyses of the HPV oncogenes E6 and E7 (Supplementary Fig. 1a). Consistent with previous reports [32–39], HPV(-) WSU12 and UM19 cells were more resistant to radiation treatment than HPV( + ) UP090 and UP154 cells (Fig. 1a). Upon radiation treatment, apoptotic cell death was evident in HPV( + ) HNSCC cells as determined by caspase activation (Fig. 1b) whereas no apoptosis was observed in HPV(-) HNSCC cells.

Next, we examined autophagic activity by monitoring the synthesis of LC3 and its lipidation to LC3-II. Immunoblotting showed that the expression level and lipidation of LC3 were higher in HPV(-) HNSCC cells in comparison with HPV( + ) HNSCC cells (Fig. 1c), suggesting that HPV inhibits autophagy in host cells through an unknown mechanism. A strong accumulation of LC3-II was observed in HPV(-) HNSCC cells treated with bafilomycin A1, an inhibitor of the lysosomal proton pump V-ATPase that inhibits autophagosome–lysosome fusion (Fig. 1d, e). These results indicate that the increases of LC3-II in HPV(-) HNSCC cells are indeed due to enhanced autophagic flux which was not observed in HPV( + ) HNSCC cells (Fig. 1d, e). To confirm differential autophagic flux between HPV(-) and HPV( + ) HNSCC in vivo, we visualized the targeting of p62^+^ to LC3^+^ autophagic membranes by the proximity ligation assay (PLA) that detects the colocalization of two proteins within the distance of 40 nm. Using antibodies against p62 and LC3 the PLA readily revealed a number of p62^+^LC3^+^ autophagic membranes in HPV(-) HNSCC tissues, whereas this complex was barely detected in HPV( + ) HNSCC tissues (Fig. 1f, g). These results demonstrate that therapy-resistant HPV(-) HNSCC cells have higher levels of autophagic flux relative to HPV( + ) HNSCC cells.

### The cytosolic levels of p62 correlates with the progression of HNSCC

**Fig. 2:**
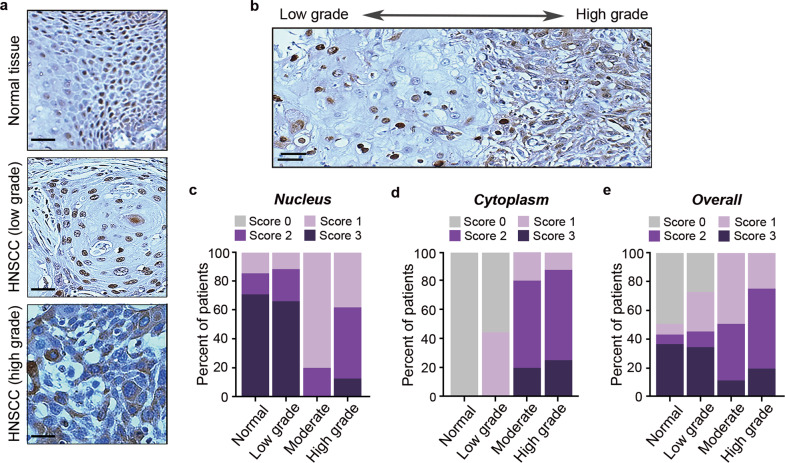
Cytosolic p62 is associated with advanced HNSCC. **a** Immunohistochemical analysis of p62 in normal tissue (top), low grade (middle), and high grade (bottom) HNSCC. Scale bar, 50 μm. **b** Immunohistochemical analysis of cytosolic and nuclear p62 localization corresponding to tumor progression. Scale bar, 50 μm. **c** Frequency of nuclear p62 in normal tissues and low, moderate, and high-grade HNSCC as assessed by expression levels (score 0–3), analyzed by Chi-square test, *p* = 0.025. **d** Frequency of cytoplasmic p62 in normal tissues and low, moderate, and high-grade HNSCC as assessed by expression levels (score 0–3), analyzed by Chi-square test, *p* < 0.001. **e** Frequency of overall p62 in normal tissues and low, moderate, and high-grade HNSCC as assessed by expression levels in both nucleus and cytoplasm (score 0–3), analyzed by Chi-square test, *p* = 0.001.

In autophagy, cargoes are selectively recognized and collected by autophagic cargo receptors such as p62 and other Sequestosome-like receptors (SLRs) [42]. Recent studies have suggested a functional and/or progonstic significance of cytosolic vs. nuclear p62 in tumor progression and therapeutic responses [43, 44]. We therefore examined the subcellular localization of p62 in HNSCC tissues. The specificity of p62 antibody was confirmed using control and p62 knockdown HPV(-) WSU cells (Supplementary Fig. 1b). The staining intensity of nuclear and cytoplasmic p62 in HNSCC tissues was evaluated by a pathologist and scored on a scale of 0 to 3. Typical immunostaining patterns of p62 in normal tissues, HPV(-) low grade, and HPV(-) high-grade HNSCC are shown in Fig. 2a. Interestingly, cytoplasmic translocation of p62 was associated with disease progression within the same patient tissue (Fig. 2b). An analysis of 7 normal tissue, 9 low grade, 5 moderate, and 8 high-grade HNSCC specimens showed that p62 was almost exclusively found in the nucleus of normal tissues and low-grade HNSCC (Fig. 2c). However, the localization of p62 gradually shifted from the nucleus to the cytosol as the tumors advanced into moderate and high-grade HNSCC (Fig. 2c, d). The overall expression levels of p62 was also associated with higher tumor grade (Fig. 2e). Collectively, these results showed association between the cytosolic localization of p62 and HPV(-) HNSCC tumor progression, consistent with a previous report that increased LC3 puncta, high cytoplasmic p62, and low nuclear p62 expressions are associated with poor prognosis and aggressive clinicopathologic features of oral squamous cell carcinoma (OSCC) [43].

### Synthetic small-molecule ligands of p62 induce autophagic flux in HPV(-) HNSCC cells

**Fig. 3:**
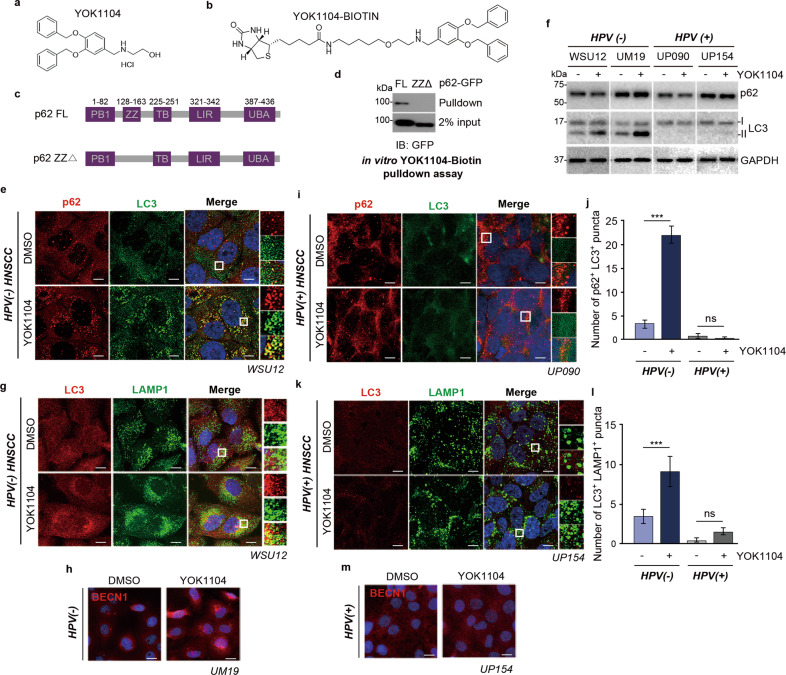
p62 ligand YOK1104 induces autophagy in radioresistant HPV(-) HNSCC. **a**, **b** Chemical structures of free or biotinylated p62-ZZ small-molecule ligand YOK1104. **c** Schematic illustration of full-length and ZZ domain-deleted mutant p62 constructs. **d** In vitro YOK1104-Biotin pulldown assay using full-length p62-GFP or ΔZZ-p62-GFP proteins, purified from HEK293 cells after transient transfection of those expression vectors. **e** Immunostaining of p62 (red) and LC3 (green) in WSU12 HPV(-) HNSCC treated with vehicle control (DMSO) or 5 μM YOK1104. Scale bar: 10 μm. **f** Immunoblot analysis of p62, LC3, or GAPDH with or without YOK1104 treatment. **g** Immunostaining of LC3 (red) and LAMP1 (green) in WSU12 HPV(-) HNSCC treated with vehicle control (DMSO) or 5 μM YOK1104. Scale bar: 10 μm. **h** Immunostaining of Beclin1 (red) in UM19 HPV(-) HNSCC treated with vehicle control (DMSO) or 5 μM YOK1104. Scale bar: 20 μm. **i** Immunostaining of p62 (red) and LC3 (green) in UP090 HPV( + ) HNSCC treated with vehicle control (DMSO) or 5 μM YOK1104. Scale bar: 10 μm. **j** Quantification of colocalization (yellow) of p62 with LC3 in **e** and **i** (*n* = 20 cells) (***p < 0.001, ns non-significant). **k** Immunostaining of LC3 (red) and LAMP1 (green) in UP154 HPV( + ) HNSCC treated with vehicle control (DMSO) or 5 μM YOK1104. Scale bar: 10 μm. **l** Quantification of colocalization (yellow) of LC3 with LAMP1 in **g** and **k** (*n* = 20 cells) (***p < 0.001, ns: non-significant). **m** Immunostaining of Beclin1 (red) in UP154 HPV( + ) HNSCC treated with vehicle control (DMSO) or 5 μM YOK1104. Scale bar: 20 μm.

We previously developed a small-molecule ligand, YOK1104, to the ZZ domain of p62 that induces self-polymerization of p62 and p62 interaction with LC3 on autophagic membranes [22]. Given the correlation between cytoplasmic p62 and HNSCC progression and the association between autophagic flux and radioresistance, we tested whether YOK1104 can modulate radiosensitivity of HPV(-) HNSCC cells. We first confirmed that YOK1104 binds to the ZZ domain of p62 using streptavidin pulldown assay with biotinylated YOK1104 (Fig. 3a–d). Next, we tested whether YOK1104 can affect p62-mediated autophagic flux in HPV(-) and HPV( + ) HNSCC cells. Immunofluorescence analyses of HPV(-) HNSCC cells treated with YOK1104 revealed a drastic increase in the formation of cytosolic p62 and LC3 puncta, which showed strong colocalization to form p62^+^LC3^+^ double positive cytosolic puncta (Fig. 3e). The formation of p62^+^LC3^+^ puncta was associated with the increases in the synthesis and lipidation of LC3 (Fig. 3f) as well as the colocalization of LC3 puncta with the cytosolic puncta positive for LAMP1, a lysosomal marker (Fig. 3g). Consistent with increased autophagic flux, YOK1104 enhanced the formation of cytosolic puncta positive for Beclin1, indicative of enhanced autophagosomal nucleation (Fig. 3h). These results show that the small-molecule ligand YOK1104 increases the activity of p62 in macroautophagy, autophagosome biogenesis, and autophagic flux in HPV(-) HNSCC cells. In contrast, YOK1104 exerted no such autophagy-inducing activity in HPV( + ) HNSCC cells as evidenced by the minimal levels of LC3-II (Fig. 3f). Likewise, neither the formation of cytosolic puncta positive for LC3 (Fig. 3i, j), the colocalization of LC3^+^ autophagosomes with LAMP1^+^ lysosomes (Fig. 3k, l) nor Beclin1 (Fig. 3m) was found in HPV( + ) HNSCC cells treated with YOK1104. These results show that YOK1104 induces p62-dependent autophagy in HPV(-) HNSCC but not in autophagy-defective HPV( + ) HNSCC cells.

### YOK1104 enhances radiosensitivity of therapy-resistant HPV(-) HNSCC cells via apoptosis induction in a p62-dependent manner

**Fig. 4:**
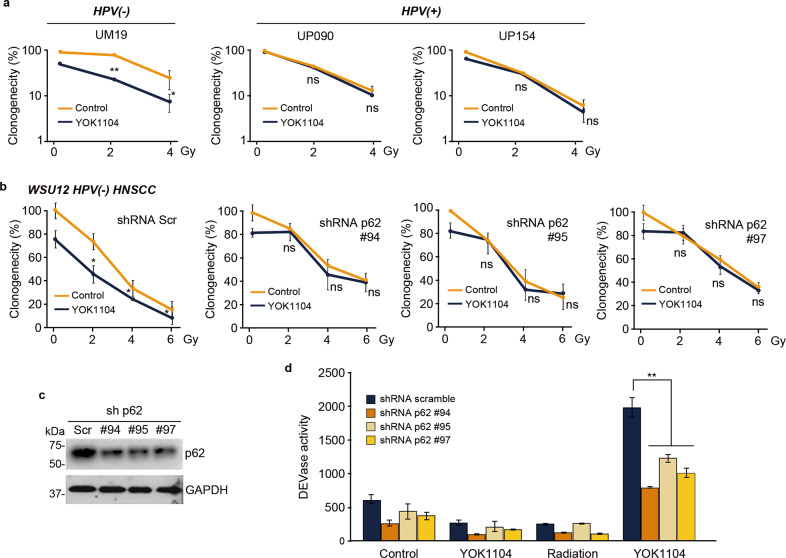
YOK1104-mediated p62 activation promotes radiotoxicity in HPV(-) via caspase activation in a p62-dependent manner. **a** Clonogenic cell survival assay of HPV(-) HNSCC cell line UM19 and HPV( + ) lines (UP090 and UP154) in the absence or presence of 5 μM YOK1104 at the indicated radiation doses (*p < 0.05, **p < 0.01, ns non-significant). **b** Clonogenic cell survival assay of control and p62 KD WSU12 cells in the absence or presence of 5 μM YOK1104 at indicated radiation doses. **c** Immunoblot analysis of p62 in HPV(-) WSU12 cells transfected with control (Scramble) or three independent vectors targeting p62 mRNA (sh p62-(#94, #95, #97). **d** Caspase-3 (DEVDase)-like activity was measured in control and p62 KD (#94, #95, #97) WSU12 cells treated with 5 μM YOK1104 and/or 6 Gy XRT (*p < 0.05, ns non-significant).

Accumulating evidence suggests that autophagy is associated with therapy-resistance [8, 9]. Besides the role of p62 in autophagy, recent studies demonstrated multi-functions of p62 including its role in caspase-8 activation [24, 25]. Here, we examined whether YOK1104 activation of p62 further increases therapy-resistance via its induction of autophagic flux or by converting its signal to programmed cell death. To this end, we first determined the effects of YOK1104 treatments on radiotherapy-induced cytotoxicity in HNSCC cells. YOK1104 rendered intrinsically radio-resistant HPV(-) UM19 and WSU12 cells sensitive to ionizing radiation (Fig. 4a, b). Notably, the radio-sensitizing efficacy of YOK1104 on HPV(-) WSU12 and UM19 cells was achieved at a dose as low as 2 Gy, a clinically relevant dose. Unlike HPV(-) HNSCC cells, YOK1104 had little effect on survival of autophagy-defective HPV( + ) UP090 and UP154 cells (Fig. 4a). Moreover, the efficacy of YOK1104 was higher in UM19 cells with higher autophagic flux compared to WSU12 cells (Fig.1d and Supplementary Fig. 1c–e). These results suggest a positive correlation between the ability of YOK1104 to promote radiotoxicity and the levels of cells’ intrinsic autophagic flux. To confirm that YOK1104-mediated cytotoxicity is dependent of p62, WSU12 cells were engineered to stably express shRNA against p62 (Fig. 4c). As shown in Fig. 4b, p62 knockdown virtually abolished the ability of YOK1104 to enhance radiotoxicity, demonstrating the specificity of YOK1104 to p62.

Next, we determined whether YOK1104-enhanced radiotoxicity in HPV(-) HNSCC cells involves apoptotic cell death. Neither YOK1104 treatment nor ionizing radiation up to 6 Gy alone was sufficient to activate DEVDase, reflecting activation of pro-caspases 3 and 7 (Fig. 4d). However, combined treatments with radiation and YOK1104 induced caspase activity, which was effectively counteracted by p62 knockdown (Fig. 4d). These results suggest that YOK1104-activated p62 induces apoptotic cell death in intrinsically therapy-resistant and apoptosis-defective HNSCC cells upon ionizing radiation treatment.

### YOK1104-activated p62 induces the formation of aggresome-like caspase-8 complex

**Fig. 5:**
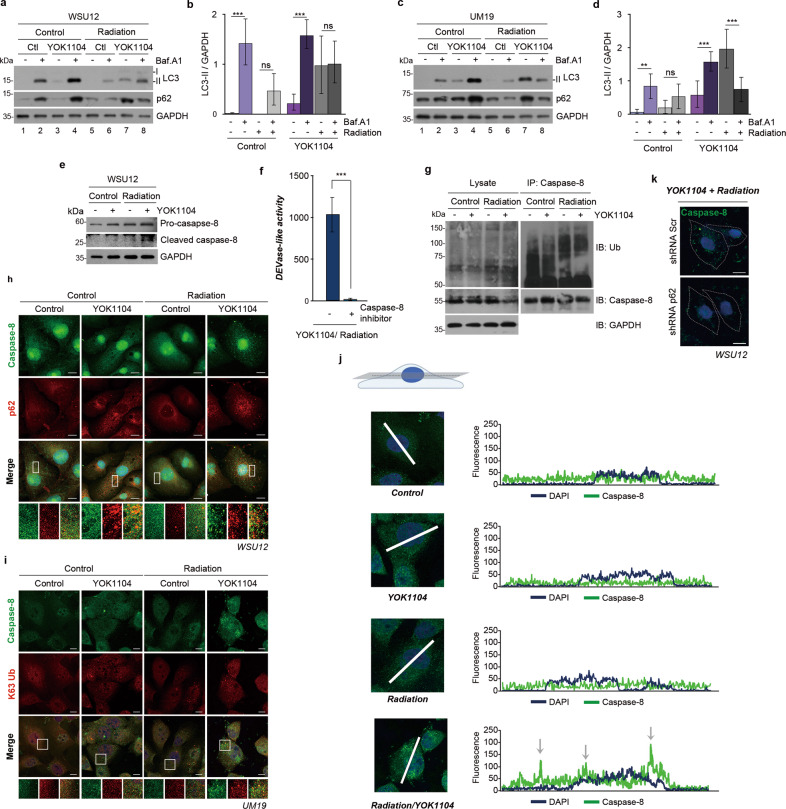
YOK1104-activated p62 sequesters ubiquitinated caspase-8 into aggresome-like structures in response to irradiation. **a** Autophagic flux assay using WSU12 cells treated with 5 μM YOK1104 (12 h) and/or 500 nM bafilomycin A1 (6 h) with or without irradiation at 6 Gy. **b** Quantification of LC3-II band intensity of **a**. Error bars represent means ± SD from three independent experiments (***p < 0.001, ns non-significant). **c** Autophagic flux assay using UM19 cells treated with 5 μM YOK1104 (12 h) and/or 500 nM bafilomycin A1 (6 h) with or without irradiation at 6 Gy. **d** Quantification of LC3-II band intensity of **c**. Error bars represent means ± SD from three independent experiments (**p < 0.01, ***p < 0.001, ns non-significant). **e** Immunoblot analysis of caspase-8 of WSU12 cells in the presence or absence of 5 μM YOK1104 with or without irradiation at 6 Gy. **f** Caspase-3/7 (DEVDase)-like activity was measured in WSU12 cells treated with 5 μM YOK1104 and 6 Gy XRT in the absence or presence of caspase-8 inhibitor (20 μM, 25 h). ***p < 0.001. **g** Immunoblot analyses of ubiquitinated proteins and caspase-8 using total WSU12 cell lysates (left panel) or caspase-8 immunoprecipitates (right panel) after treatment with 5 μM YOK1104 with or without irradiation at 6 Gy. **h** Immunostaining of caspase-8 (green) and p62 (red) in WSU12 HPV(-) HNSCC treated with vehicle control (DMSO) or 5 μM YOK1104 with or without irradiation at 6 Gy. Scale bar: 10 μm. **i** Immunostaining of caspase-8 (green) and K63-linked ubiquitin (red) in UM19 HPV(-) HNSCC treated with vehicle control (DMSO) or 5 μM YOK1104 with or without irradiation at 6 Gy. Scale bar: 10 μm. **j** Analysis of subcellular localization of caspase-8 (green) using a single focal plane at the middle of the nucleus (DAPI, blue). **k** Immunostaining of caspase-8 (green) in control and p62 KD WSU12 HPV(-) HNSCC treated with 5 μM YOK1104 and irradiation at 6 Gy. Scale bar: 10 μm.

We investigated whether pharmacological activation of p62 induces apoptotic cell death through the modulation of autophagic flux and/or caspase-8 in HPV(-) HNSCC cells upon irradiation. As expected, YOK1104 treatment enhanced autophagic flux in HPV(-) HNSCC cells as indicated by increased LC3 lipidation in the presence of bafilomycin A1 (Fig. 5a–d, lanes 4 vs. 3). It should be noted that immunoblots with shorter exposure time are shown to examine the accumulation of LC3-II and p62 in the presence of bafilomycin following treatments with YOK1104 and/or radiation. Strikingly, such autophagic flux was drastically reduced by irradiation (Fig. 5a–d, lanes 6 vs. 5). YOK1104-induced LC3-II and p62 accumulations, detected by bafilomycin treatment, was decreased upon irradiation (Fig. 5a–d, lanes 8 vs. 4). This indicates that the autophagy system is downregulated by co-treatment with YOK1104 and radiation. Thus, the p62 ZZ ligand YOK1104-promoted radiotoxicity is unlikely due to its sudden induction of autophagic flux at a toxic level.

Caspase-8 is an initiator caspase, pivotal in the extrinsic pathway of apoptosis [45, 46]. Death ligand binding to its cognate receptors on the plasma membrane induces the formation of the death-inducing signaling complex (DISC) where caspase-8 is recruited and activated [45, 46]. The E3 ligase Cullin3, that is associated with DISC, polyubiquitinates caspase-8 which subsequently interacts with the UBA domain of p62. These interactions promote aggregation and full activation of caspase-8. Subsequently, p62 transports the activated caspase-8 to cytosolic ubiquitin-rich aggresomes, which in turn induce apoptotic signaling pathways [25, 47]. Here, we examined whether YOK1104-activated p62 in combination with ionizing radiation activates the ubiquitinated caspase-8, similarly to caspase-8 activation in the extrinsic pathway. Immunoblot analyses detected an active (cleaved) form of caspase-8 in apoptosis-prone HPV( + ) UP154 cells upon irradiation (Supplementary Fig. 2a), but not in apoptosis-resistant HPV(-) WSU12 cells (Fig. 5e). While YOK1104 treatment alone had no effect on caspase-8 activation, co-treatment with radiation resulted in caspase-8 activation in HPV(-) HNSCC cells (Fig. 5e). Moreover, caspase3/7 activity induced by co-treatment was abolished in the presence of caspase-8 inhibitor (Fig. 5f). These results indicate the functional significance of caspase-8 as an initiator of caspase cascade upon YOK1104 and radiation treatments.

Next, we monitored the ubiquitination of caspase-8 in HPV(-) WSU12 cells treated with irradiation and/or YOK1104. The ubiquitination of caspase-8 was prominent upon irradiation alone but not by YOK1104 (Fig. 5g, right panel). A control experiment for the specificity of caspase-8 immunoprecipitation is shown in Supplementary Fig. 2b. We then asked whether YOK1104-bound p62 facilitates the formation of caspase-8 aggresomes in HPV(-) HNSCC cells after irradiation. Immunostaining analyses of caspase-8 and p62 did not detect caspase-8 aggresomes when treated with either irradiation or YOK1104 alone (Fig. 5h). Strikingly, a large number of caspase-8 aggresomes were generated by co-treatment with radiation and YOK1104 (Fig. 5h). Moreover, we detected colocalization of caspase-8 with K63-linked ubiquitin chains, further supporting the formation of caspase-8^+^ aggresomes by co-treatment with YOK1104 and radiation (Fig. 5i). Confocal microscopy revealed that caspase-8 puncta, generated after YOK1104/radiation co-treatment, were present mostly in the cytoplasm including the perinuclear region (Fig. 5j). Importantly, the formation of caspase-8 puncta was abolished when p62 was downregulated in WSU12 cells (Fig. 5k). Immunofluorescence staining analyses showed that while treatment with YOK1104 alone increased LC3^+^p62^+^ puncta, co-treatment with YOK1104 and radiation reduced LC3^+^p62^+^ puncta and increased caspase-8^+^p62^+^ puncta (Supplementary Fig. 2c–e). Taken together, these results demonstrate that while YOK1104 promotes autophagic flux, co-treatment with radiation exerts its cytotoxic effect by reducing autophagic flux and, in parallel, inducing apoptotic cell death mediated by caspase-8 and p62.

**Fig. 6:**
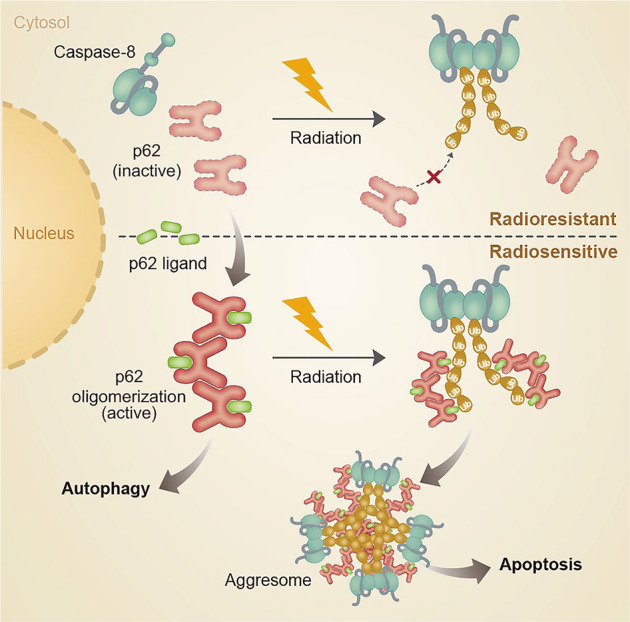
A graphical illustration of p62-dependent caspase-8 activation upon irradiation. Synthetic p62-ZZ ligands, such as YOK1104, induce p62 self-oligomerization, its interaction with LC3, and autophagosome biogenesis. Ionizing radiation treatment induces ubiquitination of caspase-8. A synthetic ligand-activated p62 via its ZZ domain promotes aggregation of ubiquitinated caspase-8 into aggresomes-like structures leading to apoptosis.

In conclusion, this study suggests that irradiation induces ubiquitination of caspase-8 by an as-yet-unknown mechanism, and ubiquitination of caspase-8 itself is insufficient to interact with p62 for the formation of aggresome. Importantly, pharmacologic activation and oligomerization of p62 provides a focal point for caspase-8 aggregation leading to caspase-8 activation (Fig. 6). Thus, we propose that small-molecule ligands to the p62 ZZ domain have therapeutic potential as radiosensitizers in intrinsically apoptosis-resistant HPV(-) HNSCC.

## Discussion

In this study, we investigated the mechanisms underlying radioresistance in HPV(-) HNSCC and examined the potential of p62 as a druggable target to improve the efficacy of radiation therapy. To this end, we first characterized the distinct responses of HPV( + ) vs. HPV(-) HNSCC to ionizing radiation and observed that the latter were not only less susceptible to radiotoxicity but also resistant to caspase activation. HPV(-) HNSCC exhibited both drastically greater levels of autophagosomes and their accelerated turnover. In association with this, our study suggests that the cytosolic level of p62 and cellular level of autophagy flux correlate with the radioresistance and the progression of HPV(-) HNSCC. We explored whether this pro-survival autophagic pathway can be targeted by novel therapies. A small-molecule ligand to the ZZ domain of p62, termed YOK-1104, was successfully used to induce p62 self-oligomerization, autophagic sequestration of its cargoes, and autophagosome biogenesis in HPV(-), in sharp contrast to HPV( + ) HNSCC. Such structural and functional activation of p62 by a synthetic ligand sensitized HPV(-) HNSCC to radiation-induced radiotoxicity. Mechanistically, irradiation of HPV(-) HNSCC results in ubiquitination of caspase-8, which appears to be a prerequisite but insufficient for its interaction with p62. Importantly, pharmacologic activation of p62 facilitates caspase-8^+^p62^+^ aggresome-like structures for the activation of the subsequent apoptotic signaling cascade. Our finding that synthetic ligand-mediated p62 activation promotes radiotoxicity via induction of apoptosis in cells with high autophagic flux is particularly significant since this approach may induce malignant cancer-specific cell death with less normal cell toxicity.

Interestingly, HPV(-) HNSCC cells not only exhibited higher basal degrees of autophagy flux and p62–LC3 interaction as opposed to their HPV( + ) counterparts, but also were more readily responsive to p62-ZZ ligand-mediated autophagy induction. Our results showed that synthetic activation of p62 via its ZZ domain primes HPV(-), but not HPV( + ), HNSCC for radiation-induced cell death by sequestering ubiquitinated caspase-8 into aggresome-like structures. Failure of p62 activation in HPV( + ) cells would hinder the self-oligomeric activity of p62 and the ability to sequester its cargoes, including caspase-8. Given the significance of p62-regulated ER stress signaling in the regulation of both autophagy and programmed cell death, it would be of importance to investigate functional relationship between radiosensitivity and misregulation of p62 and/or the N-degron pathway in the future. Interestingly, photodynamic therapy (PDT) directed at the ER/mitochondria using benzoporphyrin derivative (BPD) synergized with YOK1104 for caspase activation whereas PDT using NPe6, a selective photosensitizer for lysosomes, failed to do so (Supplementary Fig 3).

Upon death signals, the E3 ligase Cullin3 polyubiquitinates autoprocessed caspases-8 catalytic domain fragments, which in turn recruits p62 to the ubiquitinated chains. p62 transports the liberated catalytic domain of casepase-8 to ubiquitin-rich “aggresomes” in the cytosol [25]. A pertinent question in our study is why ubiquitinated caspase-8 fails to form aggresomes with p62 in HPV(-) HNSCC cells upon irradiation. We surmise that in the absence of adaptor proteins such as FADD in DISC, monomers of caspase-8 and p62 fail to interact with each other in the formation of aggresome. Importantly, our study demonstrated that YOK1104-induced oligomerization and activation of p62 are sufficient to initiate the formation of caspase-8^+^p62^+^ puncta upon co-treatment with radiation even in the absence of death receptor activation. It would be of importance to determine the subcellular location where YOK1104-activated p62 oligomers initiate aggresome formation with caspase-8^+^ upon irradiation. It also remains to be seen how p62-mediated aggregation of caspase-8 activates downstream executioner caspases. One possibility is that p62-associated caspases-8^+^ aggresomes prolong the half-life of polyubiquitinated caspase-8 beyond the necessary threshold for the activation of downstream executioner caspases, instead of being targeted to the proteasome, as previously suggested [25]. Taken together, our study provides molecular insights into the functional interplay between autophagy and programmed cell death pathways regulated by p62. We, thus, propose that p62 is a druggable target for the manipulation of sensitivity to death stimuli in cancer cells, typically resistant to both intrinsic and extrinsic cell death stimuli (Supplementary Fig. 4). In addition to cancer therapeutics, our findings may have broad biomedical applications since imbalance between autophagy and apoptosis are often involved in many human diseases.

## Supplementary information

Supplementary information

Supplementary Figure 1

Supplementary Figure 2

Supplementary Figure 3

Supplementary Figure 4

## Data Availability

All data is available in the manuscript file and its supplementary information file or available upon request.
