# Supplementary material for: p62/SQSTM1-induced caspase-8 aggresomes are essential for ionizing radiation-mediated apoptosis
Source: Cell Death Dis. 2021 Oct 25;12(11):997. doi: 10.1038/s41419-021-04301-7 (PMC8546074; doi:10.1038/s41419-021-04301-7)
Supplement: Supplementary file 1 — Supplementary information [file 41419_2021_4301_MOESM1_ESM.docx]

**Supplementary Figure Legends**

**Supplementary Figure 1.** **Characterization of HPV status in HNSCC cell lines and the effects of YOK1104 treatment on the radiosensitivity of HPV(-) and HPV(+) HNSCC cells**

(**a**) RT-PCR analysis of HPV oncogenes E6 and E7 expression in various HNSCC cell lines. (**b)** Imunohistochemical analysis of WSU12 cells under shRNA-mediated p62 knockdown. Scale bar: 30 μm. (**c, d, e**) Clonogenic cell survival assay of WSU12, UM19, UP090 cell lines upon ionizing radiation at indicated doses in the presence of vehicle control (DMSO) or YOK1104 (5 μM, 2 days). Cell survival fraction is shown on a linear scale.

**Supplementary Figure 2. Radiation treatment reduces the level of autophagy in HPV(-) HNSCC, and YOK1104 induces apoptotic cell death mediated by caspase-8 and p62**

(**a**) Immunoblot analysis of caspase-8 of UP154 cells in the presence or absence of 5 μM YOK1104 with or without irradiation at 6 Gy. (**b**) Immunoblot analysis of caspase-8 using total lysates or using control IgG and caspase-8 IP products. (**c**) Immunofluorescence staining of LC3, caspase-8 and p62 in WSU12 cells in the presence or absence of 5 μM YOK1104 with or without irradiation at 6 Gy. (**d**) Quantification of colocalization (purple) of p62 with LC3 in **c** (**p* < 0.01, ***p* < 0.001, ns: non-significant). (**e**) Quantification of colocalization (yellow) of p62 with Caspase-8 in **c** (**p* < 0.01, ***p* < 0.001, ns: non-significant).

**Supplementary Figure 3. YOK1104 promotes caspase activation together with ER/mitochondria-targeted photodynamic therapy (PDT), but not with lysosome-targeted PDT**

DEVDase (caspase 3-like) activity assay in WSU12 in the presence or absence of 5 μM YOK1104, and with or without PDT directed at the ER/mitochondria using 0.5 μM benzoporphyrin derivative (BPD) at 22.5 mJ/cm^2^ (**a**) or at the lysosome using 20 μM NPe6 at 30 mJ/cm^2^ (**b**).

**Supplementary Figure 4. HPV(-) HNSCC cells are resistant to both intrinsic and extrinsic apoptotic stimuli compared to HPV(+)HNSCC cells**

(**a**) DEVDase (caspase 3-like) activity assay in WSU12, UM19, UP090 and UP154 cell lines treated with stauroporine (0.5 μM), known to induce mitochondrial intrinsic apoptotic pathway. (**b**) Caspase 3 activity assay of WSU12, UM19, UP090 and UP154 cell lines treated with TRAIL (100 ng/mL), known to induce extrinsic apoptotic pathway involving DISC formation.
