# Supplementary figures and images for: p62/SQSTM1-induced caspase-8 aggresomes are essential for ionizing radiation-mediated apoptosis
Source: Cell Death Dis. 2021 Oct 25;12(11):997. doi: 10.1038/s41419-021-04301-7 (PMC8546074; doi:10.1038/s41419-021-04301-7)

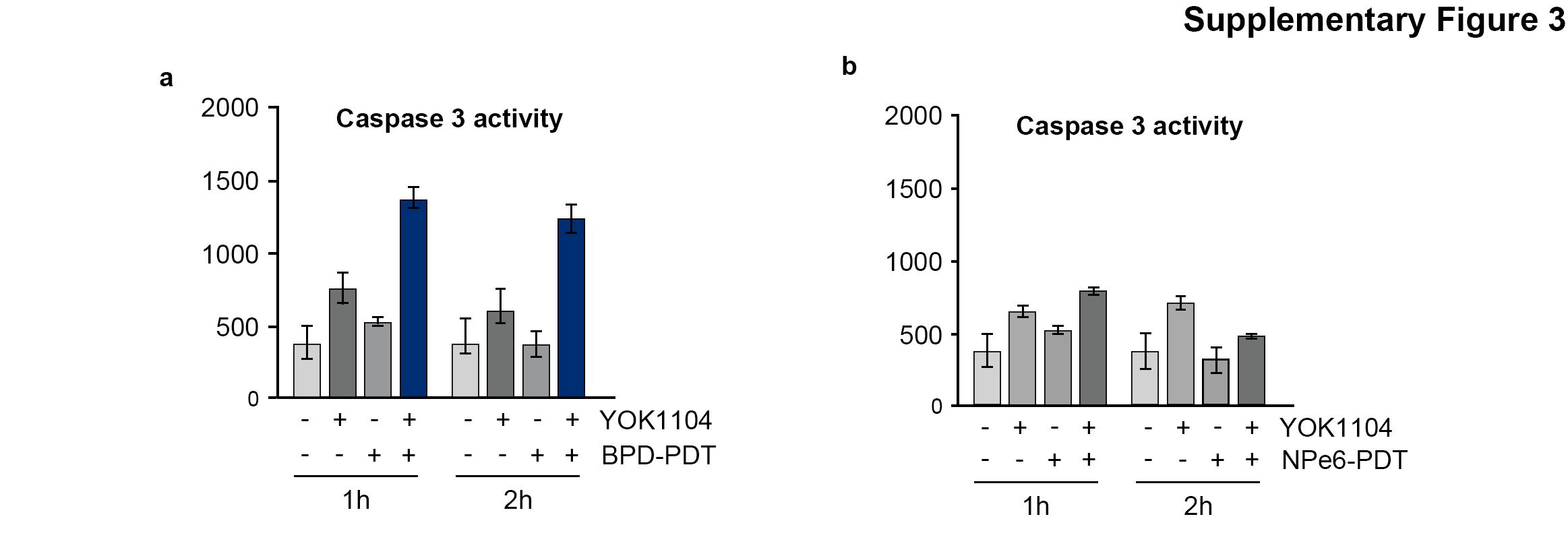

Supplement: Supplementary file 4 — Supplementary Figure 3 [file 41419_2021_4301_MOESM4_ESM.tif]
